# Supplementary material for: Dynamic encoding of social threat and spatial context in the hypothalamus
Source: eLife. 2020 Sep 21;9:e57148. doi: 10.7554/eLife.57148 (PMC7505658; doi:10.7554/eLife.57148)
Supplement: Supplementary file 1. — Territory includes both Home and Far chamber cells. [file elife-57148-supp1.docx]

| **Mouse** | **Assessment** | **Defense** | **Flight** | **Sniff** | **Attack** | **Territory** |
| --- | --- | --- | --- | --- | --- | --- |
| 1 | 41 | 39 | - | 55 | 55 | 44 |
| 2 | 57 | 58 | 58 | 61 | 61 | 61 |
| 3 | 43 | 30 | 44 | 46 | - | 44 |
| 4 | 59 | 60 | - | 71 | 72 | 62 |
| 5 | 35 | 36 | - | 41 | 41 | 35 |
| 6 | 53 | 53 | 53 | - | - | 54 |
| 7 | 38 | 43 | 43 | 36 | 37 | 43 |
| **Total** | **326** | **319** | **198** | **310** | **266** | **343** |
